# New Jersey

**Published:** 1899-05

**Authors:** S. Lockwood

**Affiliations:** Secretary


### SOCIETY PROCEEDINGS.

### VETERINARY MEDICAL SOCIETY OF NEW JERSEY.

THE Association held its regular meeting at the Arlington Hotel, Newark, at 10 A.M., May 11, 1899, with the following members present: Drs. L. P. Hurley, J. W. Hawk, W. Runge, J. Kehoe, B. F. King, J. M. Everitt, M. M. Stage, W. B. E. Miller, R. O. Hasbrouck, H. W. Read, W. Gall, A. D. Edwards, A. W. Axford, H. Exton, and S. Lockwood. Dr. T. E. Budd, of Orange, was elected a member. Five names were proposed for membership, to be voted on at the next regular meeting.

Each member present reported cases in his practice, with treatment and results, making it one of the best meetings in the history of the Association. As it was time to adjourn, the reading of papers was postponed to next meeting. Adjourned at five o'clock, to meet at same place in September.

S. LOCKWOOD, V.S.,
Secretary.

